# Computer-Aided Saturation Mutagenesis of *Arabidopsis thaliana Ent*-Copalyl Diphosphate Synthase

**DOI:** 10.1007/s12539-019-00342-x

**Published:** 2019-07-15

**Authors:** Piotr Szymczyk, Grażyna Szymańska, Anna Lipert, Izabela Weremczuk-Jeżyna, Ewa Kochan

**Affiliations:** 1 Department of Pharmaceutical Biotechnology, Faculty of Pharmacy, Medical University of Łódź, 90-151 Lodz, Poland; 2 Department of Biology and Pharmaceutical Botany, Faculty of Pharmacy, Medical University of Łódź, 90-151 Lodz, Poland; 3 Department of Sports Medicine, Medical University of Łódź, 92-213 Lodz, Poland

**Keywords:** In silico saturation mutagenesis, *Ent*-copalyl diphosphate synthase, Enzyme design

## Abstract

*Ent*-copalyl diphosphate synthase controls the biosynthesis of gibberellin plant hormones, which in turn coordinate the expression of numerous enzymes. Some gibberellin-dependent genes encode enzymes coordinating the biosynthesis of tanshinones: diterpene derivatives with broad medical applications. New biotechnological approaches, such as metabolic engineering using naturally occurring or mutated enzymes, have been proposed to meet the growing demand for tanshinones which is currently met by the Chinese medicinal plant *Salvia miltiorrhiza* Bunge. These mutants may be prepared by directed evolution, saturation mutagenesis or rational enzyme design. In the presented paper, 15,257 non-synonymous variants of *Arabidopsis thaliana ent*-copalyl diphosphate synthase were obtained using the SNAP2 tool. The obtained forms were screened to isolate variants with potentially improved biological functions. A group of 455 mutants with potentially improved stability was isolated and subjected to further screening on the basis of ligand–substrate affinity, and both secondary structure and active site structure stability. Finally, a group of six single mutants was obtained, which were used to construct double mutants with potentially improved stability and ligand affinity. The potential influence of single mutations on protein stability and ligand affinity was evaluated by double mutant cycle analysis. Finally, the procedure was validated by in silico assessment of the experimentally verified enzyme mutants with reduced enzymatic activity.

**Electronic supplementary material:**

The online version of this article (10.1007/s12539-019-00342-x) contains supplementary material, which is available to authorized users.

## Introduction

*Ent*-copalyl diphosphate synthase (EC 5.5.1.13) (*ent*-CPS) coordinates the supply of precursors to the biosynthesis of plant hormone gibberellins [1]. This enzyme catalyses the biosynthesis of *ent*-copalyl diphosphate from geranylgeranyldiphosphate. The crucial role in catalysis is played by the middle D residue (D379) of the D^377^XDD motif [1, 2], which in the case of *Arabidopsis thaliana ent*-CPS, attacks the double carbon bond, thus creating a reactive carbocation which undergoes subsequent rearrangements [1]. X-ray crystallography studies of *A. thaliana ent*-CPS have indicated that the general acidic D379 forms a hydrogen bond with N425: the side chain carboxamide group of N425 helps orient the catalytic OD2 toward the active site by directly interacting with the OD1 of D379. Another functionally important aa residue adjacent to D379 is T421 [1, 3]. The side chain hydroxyl group of T421 is believed to stabilize the acid side chain of D379 by a water-bridged hydrogen bond [1, 3]. In addition, the Grotthuss diffusion-based reprotonation of D379 in the catalytic cycle is facilitated by two channels that exist separately from the main entrance to the active site; they have been found around R340, D336 and D503. The directed mutagenesis of these aa residues together with H331 and D377 resulted in a significant drop of enzymatic activity, confirming their pivotal role in the catalytic reaction [1, 3, 4].

Besides *ent*-CPS, plants usually possess numerous genes encoding for another enzyme: copalyl diphosphate synthase (CPS) (EC 5.5.1.12) [5–8], which produces the (+)-copalyl diphosphate used in the biosynthesis of numerous diterpenoids [9–11]. Among these are the tanshinones, which demonstrate a range of antibacterial, anticancer and anti-inflammatory properties [8, 9, 12]. Although these tanshinones have traditionally been obtained from the Chinese herbal plant Danshen (Tanshen), also known as red sage (*Salvia miltiorrhiza* Bunge) [12], demand has grown considerably in recent years; this fact, combined with the limited area available for plant cultivation and increasing levels of soil pollution, has prompted the need to develop new biotechnological methods to increase production. One of the most promising approaches involves the use of metabolic engineering based on the increased expression of genes encoding for enzymes of crucial importance for tanshinone biosynthesis in transformed *S. miltiorrhiza* plants [11, 13, 14]. Alternatively, *trans*-factors controlling the expression of particular pathway genes can be overexpressed in the transformed plant in a coordinated way [15, 16].

Although *ent*-CPS and (+)-CPS produce precursors of different metabolites, i.e., gibberellins or tanshinones, their functions are connected. Plant hormone gibberellins regulate expression by a complex route including feedback regulation and modulation of the genes used in biosynthesis, including those from the tanshinone biosynthesis pathway [17]. Gibberellin-dependent tanshinone production can be increased by the activity of participating *trans*-factors, such as the gibberellin-responsive binding factors *Sm*GRAS1-5 [18]. Therefore, tanshinone yield could be boosted by increasing endogenous hormone biosynthesis induced by improved *ent*-CPS activity in transformed plants. This could be realized by overexpression of native *ent*-*CPS*, or of a redesigned form with improved catalytic properties. Such an enzyme modification may be pursued experimentally by directed evolution [19], a tedious approach, or by rational enzyme redesign [20–23]: an approach that uses structure data, site-directed mutagenesis, homology modeling and protein biophysical properties to create smaller variant libraries than those observed in directed evolution experiments. These approaches may be supported by available in silico tools as SNAP or SNAP2 used to perform in silico saturation mutagenesis, i.e., the substitution of each aa residue by all remaining aa residues [24–26]. The obtained variants are characterized by a SNAP2 score varying from − 100 to 100. Although the threshold discriminating neutral and non-neutral variants is in theory zero, higher, positive threshold values can be used to allow more accurate predictions to be made of functionally important variants [26].

The presented report examines the in silico saturation mutagenesis of *A. thaliana ent*-CPS. Briefly, 15,257 variants were initially screened by the SNAP2 tool to select the forms which most significantly changed the properties of the protein. As a result, a shortlist of 455 mutants was prepared. These mutants were tested in silico on the *A. thaliana ent*-CPS structure with the docked physiology substrate geranylgeranyl diphosphate. Free folding energy, free energy of ligand binding, secondary structure or active site structure changes were evaluated to allow the number of mutants that could positively affect the enzyme activity to be further reduced. In addition, double mutants were prepared and subsequently evaluated by double mutant cycle analysis to find potential interactions between the aa residues constituting the double mutants.

## Materials and Methods

### Searching for *A. thaliana Ent*-CPS Variants Affecting Enzyme Functions

The analysis used an improved version of SNAP, known as screening for non-acceptable polymorphisms 2 (SNAP 2): a neural network-based tool outperforming other systems and their combinations (SIFT, SNAP, PolyPhen-2) in distinguishing between neutral and non-neutral variants [24–27]. The SNAP2 tool is available online at https://rostlab.org/services/snap2web (Garching/Munich, Germany). Mutant selection criteria were assigned to “effect” scores higher than 85 and a probability of at least 91%. The scores presented by SNAP2 vary between − 100 (strongly expected “neutral”) and 100 (strongly expected “effect”). Non-neutral (“effect”) mutants demonstrate altered protein function, while the “neutral” ones do not. Hecht et al. [26] propose that while scores higher than 60 are strongly indicative of the functional importance of a particular residue, higher values, of at least 80, are needed to efficiently remove false positives. Therefore, a SNAP2 score threshold of at least 85 is used in the present study [27]. In addition, all variants characterized by SNAP2 scores of at least 85 returned a probability outcome greater than 91%, i.e., the chance that the predicted effect, or its absence, is true [26]. Therefore, 91% was established as the lowest probability threshold in the study.

### Ligand Docking

A crystal structure of *A. thaliana ent*-CPS (GenBank 4LIX) with the docked un-physiology ligand (*S*)-15-aza-14,15dihydrogeranylgeranyl thiolodiphosphate was used for analysis. The resolution of the presented crystal structure was found to be 1.55 Å [3]. The 3D structure of the physiology ligand geranylgeranyl diphosphate was obtained from the PubChem database (https://pubchem.ncbi.nlm.nih.gov) (PubChem CID 5497105) and converted to a pdb-format file using the online Smiles Translator and Structure File Generator (https://cactus.nci.gov/translate). The not native ligand (*S*)-15-aza-14,15-dihydrogeranylgeranyl thiolodiphosphate was removed from the crystal structure of *A. thaliana ent*-CPS, together with the water and residual solvents. The geranylgeranyldiphosphate was docked into the active pocket of *A. thaliana ent*-CPS using Auto Dock Vina software configured with default settings [28]. The final ligand pose was selected on the grounds of the lowest ligand free energy of binding, with the aa residues of well-defined function in contact with the ligand [3]. To identify the best docking pose, the ligand free energy of binding (Δ*G*_BIND_) and ligand affinity (*K*_A_) values were calculated using the following equation:

$$\Delta G_{\text{BIND}} = \, {-} \, RT{\text{ln(}}K_{\text{A}} /C ) ,$$

where *T* is the temperature in kelvin, *C* is 1 M concentration and *R* = 8.314 J/mol/K. The selected ligand–enzyme complex was used for further analysis.

### Single Mutant Preparation and Evaluation

As a starting point, a group of 455 mutants of *A. thaliana ent*-CPS was used. This group was selected from 15,257 members of the saturation mutagenesis library on the basis of the SNAP2 score threshold provided in Sect. 2. Mutagenesis was performed by FoldX software [29], a package chosen for its ease of use and its reputation as one of best programs to evaluate the changes in the stability of protein point mutations [30, 31]. Mutants were characterized by the changes in free protein folding energy occurring after mutation:

$$\Delta \Delta G_{\text{FOLD}} = \, \Delta \Delta G_{\text{FOLD MUT}} - \, \Delta \Delta G_{\text{FOLD WT}} .$$

Mutations that cause negative changes in ΔΔ*G*_FOLD_ are generally recognized as stabilizing protein structure. In the present study, such mutations were categorized as follows: − 1.00 kcal/mol < ΔΔ*G*_FOLD_ < − 0.50 kcal/mol (minor stabilization changes), − 2.00 kcal/mol < ΔΔ*G*_FOLD_ < − 1.00 kcal/mol (moderate stabilization changes—“warm residues”) and ΔΔ*G*_FOLD_ ≤ − 2.00 kcal/mol (strong stabilization changes—“hot spots”) [32, 33]. Only mutants indicating ΔΔ*G*_FOLD_ < − 0.50 were subjected to further analysis.

Selected mutants were used to dock the physiology substrate geranylgeranyl diphosphate (GGPS) into the active site of *A. thaliana ent*-CPS using AutoDock Vina software as described in Sect. 2 [28]. The change of ligand free energy of binding ΔΔ*G*_BIND_ was calculated for each mutant as follows:

$$\Delta \Delta G_{\text{BIND}} = \, \Delta \Delta G_{\text{BIND MUT}} - \, \Delta \Delta G_{\text{BIND WT}} .$$

Quantitative ranges to discriminate between “hot”, “warm” or residues of minor importance for ΔΔ*G*_BIND_ were the same as provided for ΔΔ*G*_FOLD_ [32–34]. Only mutations indicating ΔΔ*G*_BIND_ < − 0.50 kcal/mol were used in further analyses. In addition, ligand affinity (*K*_A_) was calculated for the best mutated docking pose using the equation provided earlier in Sect. 2.

The mutants were then analysed by PsiPred software to identify potential changes in protein secondary structure [35]. Those with stable secondary structures were evaluated according to potential changes in the geometry of the ligand–enzyme complex, measured as the distance between the substrate and the aa residues known to play a role in the catalytic reaction. Analyses were performed for both ligands: (*S*)-15-aza-14,15dihydrogeranylgeranyl thiolodiphosphate present in the crystal structure of *A. thaliana ent*-CPS (GenBank 4LIX) and the same enzyme with docked GGPS as the native substrate [3]. YASARA software was applied to calculate interatomic distances [36].

### Validation of Variant Screening Methodology

The presented screening procedure was validated on known mutants with experimentally verified thermodynamic or catalytic properties. Mutants of *A. thaliana ent*-CPS with decreased catalytic function, such as H331A, H331R, D377A, D379A and D380A are relatively well characterized [2, 4], while E211A, R340A, T421A, T421S, N425A and D503A are known to have negative kinetic properties [3]. To determine whether they could be efficiently screened out as structures with potentially unfavorable catalytic or thermodynamic properties, all presented variants were evaluated with the same techniques used to analyse the single mutants, described above.

### Double Mutant Preparation and Double Mutant Cycle Analysis

The single mutants that passed all the described quality tests were used to obtain a group of double mutants. These were analysed using the same quality tests as described for the single mutants. Double mutants passing all quality tests were then characterized by double mutant cycle analysis to identify possible interactions occurring between mutated aa residues. All five types of theoretically possible interactions occurring between two mutated aa residues were defined as described by Mildvan [37].

### Statistical Analysis

A nonparametric test was chosen to analyse the data, as it did not have a normal distribution. Chebyshev’s inequality was applied to calculate the confidence intervals and minimal percentage of results within *k* standard deviations of the mean [38]. This inequality states that the following conditions occur for a random variable *X* with finite expected value *µ*, finite non-zero variance *σ*^2^ and for any real number *k* > 0:

$$Pr(|X - \mu |) \ge k\sigma ) \le 1/k^{2} .$$

The mean and standard deviation *σ* were calculated, and the results were used to calculate the number of multiplications (*k*) of standard deviation *σ* from the mean that the particular result was localized. Based on the width of the confidence interval, the probability (%) that the maximal number of a particular result was localized beyond the *k*-fold of standard deviation *σ* was obtained by Chebyshev’s inequality. For example, assuming that the confidence interval is five times larger than the *σ* of the mean value (*k* = 5), the probability that a maximal number of results is beyond this area is *p* ≤ 1/*k*^2^ (*P* ≤ 1/25; *p* ≤ 0.04). Values of *p* ≤ 0.05 are recognized as statistically significant.

Chebyshev’s inequality was applied for the statistical analysis of interatomic distances in wild-type and mutated forms of *A. thaliana ent*-CPS. Aside from the very simple and elementary applications of Chebyshev’s inequality presented in our present work, the theorem has also been applied for advanced mathematical and statistical approaches, one of which is the novel complex probability concept of Jaoude, being the sum of real and imaginary sets, extending the number of classical Kolmogorov axioms from 5 to 8 [39]. In addition to samples of known mean and variance, Chebyshev’s inequality may also be generalized to multiple dimensions, as long as the samples are independent and equally distributed [40].

## Results

### Stability of Single Mutants

SNAP2 tool allowed all non-native single variants of *A. thaliana ent*-CPS to be constructed and characterized. In total, 15,257 mutants were created by substituting each of *A. thaliana ent*-CPS 803 aa residues with 1 of 19 non-native amino acids. The obtained variants are characterized by an effect score [− 100; 100] and probability (%) of correct effect or neutral prediction. The scale [− 100; 100] ranges from a very strong neutral prediction (− 100) to a very strong effect prediction (100). To obtain results with a very strong predictive value, a score of at least 85 and probability of effect/neutral correct prediction equal to or higher than 91% were applied [26, 27]. Mutations with a high effect score are expected to significantly affect protein function. The application of these threshold scores allowed 455 *A. thaliana ent*-CPS variants to be isolated from the population of 15257 initially produced by the SNAP2 tool (Table S1).

Among the 455 tested variants, only 14 indicated a potential stabilizing effect, i.e., ΔΔ*G*_FOLD_ values lower than the threshold value of—0.50 kcal/mol (Table S2). To more precisely evaluate the potential changes of protein stability induced by the mutation, the secondary structure was analysed, as structural changes could have a negative effect on protein structure and catalytic activity [41, 42]. PsiPred software analysis identified five single mutants with such changes, with ΔΔ*G*_FOLD_ values lower than—0.50 kcal/mol (Table 1). These five mutants were removed from further analysis. Of the remaining nine mutants carried to the next stage of analysis, three were recognized as moderate stabilizing variants and two were clear “hot” mutation spots with a strong stabilizing effect. The remaining four single mutants demonstrated minor stabilizing activity.

**Table 1:** Changes in secondary structure, ligand free energy of binding and ligand affinity upon mutation of *A. thaliana ent*-CPS

Nr	Mutation	Secondary structure change and accuracy of prediction (%)	ΔΔ*G*_BIND_ (kcal/mol)	*K*_A_ (nM)
–	WT	–	− 8.91	292.88
1	**T114F**	None 100%, coil → coil	− 0.77	79.99
2	**D336L**	None 100% helix → helix	− 0.59	108.56
3	**D377L**	None, 100%, coil → coil	− 0.76	82.31
4	D377K	None, 100%, coil → coil	− 0.23	200.67
5	H391L	Change, 0% helix → other than helix and sheet	− 0.40	150.11
6	H391M	Change, 0% helix → other than helix and sheet	− 0.13	237.97
7	**G422L**	None, 100% helix → helix	− 0.54	119.32
8	G422M	None, 100% helix → helix	− 0.12	241.61
9	**S597W**	None, 100% helix → helix	− 0.60	106.92
10	**K778F**	None, 100% helix → helix	− 0.54	118.12
11	K778W	None, 100% helix → helix	− 0.44	140.55
12	H793F	Change, 0%, other than helix and heet → other than helix and sheet	− 0.52	123.21
13	H793L	Change, 0%, other than helix and heet → other than helix and sheet	− 0.61	104.43
14	H793M	Change, 0%, other than helix and heet → other than helix and sheet	− 0.61	104.43

Six selected single mutants that passed the presented quality test are marked in bold

### Changes in Ligand Free Energy of Binding Induced by Mutation

Further screening was performed based on the changes in ligand (GGPS) ΔΔ*G*_BIND_ occurring upon mutation. The mutants D377K, G422M and K778W of *A. thaliana ent*-CPS were found to demonstrate ΔΔ*G*_BIND_ values greater than—0.50 kcal/mol, and so were removed from the study (Table 1). The remaining six mutations induced only minor negative changes to ΔΔ*G*_BIND_. These six forms also showed improved affinity for the physiology ligand GGPS, ranging from 79.99 nM (T114F) to 119.32 nM (G422L). By comparison, the value for wild-type (WT) *A. thaliana ent*-CPS was 292.88 nM (Table 1).

**Fig. 1:**
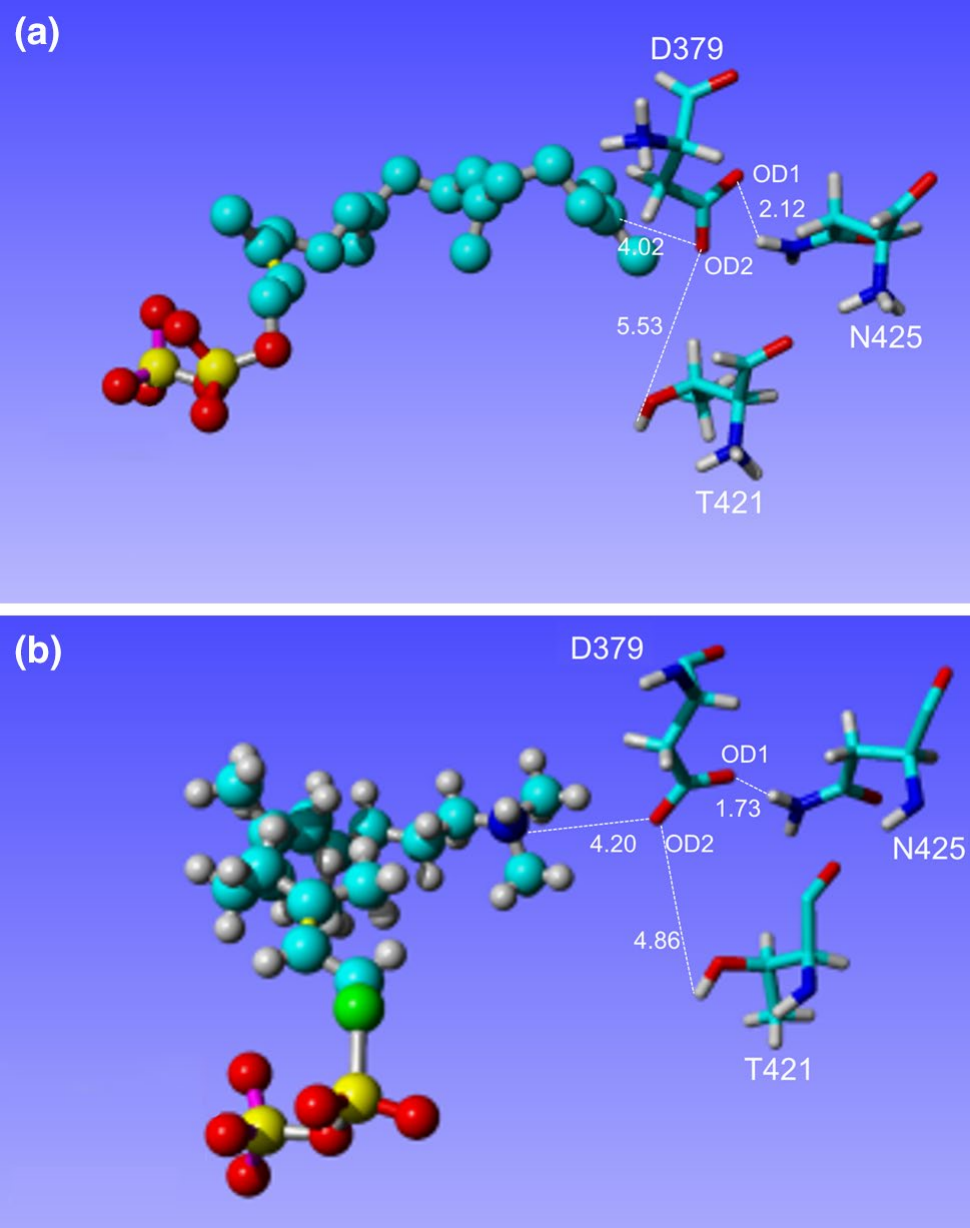
Interatomic distances between aa residues of crucial importance for catalytic reaction of *A. thaliana ent*-CPS. The *A. thaliana ent*-CPS with docked GGPS (**a**) and original enzyme structure reported by Köksal et al. [3] with (*S*)-15-aza-14,15dihydrogeranylgeranyl thiolodiphosphate (**b**) were provided

Finally, the distance between the ligand and the aa residues participating in the catalytic reaction (D379, T421, N425) was evaluated and compared to values obtained for the native enzyme. The important interaction playing a crucial role in the catalytic reaction occurs between OD2 of D379 and C15 of the GGPS ligand; this distance was found to be 4.02 Å in the protein with a docked GGPS ligand. Similarly, the distance between D379:OD2 and the nitrogen atom of (*S*)-15-aza-14,15dihydrogeranylgeranyl thiolodiphosphate, corresponding to C15 in GGPS was found to be 4.20 Å [3] (Fig. 1).

The catalytic D379:OD2 is oriented towards the substrate by a water-bridged hydrogen bond with an OH group of the side chain of T421. The distance between D379:OD2 and the T421 OH group in the enzyme with (*S*)-15-aza-14,15dihydrogeranylgeranyl thiolodiphosphate was found to be 4.86 Å [3]; however, this distance was 5.53 Å in the enzyme with docked GGPS (Fig. 1).

Another residue stabilizing D379 is N425. The carboxamide group of N425 builds a hydrogen bond with OD1 of D379, further stabilizing the orientation of D379 OD2 towards the substrate. The corresponding distances were 1.73 Å in the crystal structure and 2.12 Å in the structure with docked GGPS (Fig. 1) [3]. Therefore, the interatomic distances reported by Köksal [3] are well reproduced by *A. thaliana ent*-CPS structure with docked GGPS, confirming their biological functionality and reliability.

Based on observed reproduction of their structural relationship with the two ligands, the three presented aa residues, D379, T421 and N425, were used to calculate the distances to the ligand in the native and mutated forms (Table S3). While the distance D379:OD1-N425:HD2 was found to be shorter in WT (2.12 Å) than in the mutated variants (2.23–2.26 Å), the opposite was true for the distances between D379:OD2 and GGPS:C15 (3.24–3.32 Å in mutants compared to 4.02 Å in WT), and between D379:OD2 and T421:HG1 (5.11–5.23 Å compared to 5.53 Å in WT) (Table S3). Only differences in the distances between D379:OD2 and GGPS:C15 were statistically significant.

### Validation of the Single Mutants Screening Procedure

Due to the lack of literature data concerning the improved catalytic properties of *A. thaliana ent*-CPS, the mutants that negatively affect catalytic functions were also studied. These mutants were screened as described in Sect. 2. None of the 11 tested mutants passed the threshold SNAP2 scores (85% effect and 91% probability) (Table 2), and only 1 of them passed the ΔΔ*G*_FOLD_ test (D380A) (Table 2). The secondary structure changes measured by PsiPred software were observed in only 1 mutant (E211A); however, the ΔΔ*G*_BIND_ values allowed 3 of the 11 mutants to be removed (Table 2).

**Table 2:** Validation of mutant screening method applied to eleven *A. thaliana ent*-CPS variants of impaired enzymatic activity

Nr	Mutation	Effect score [− 100; 100]	Probability (%)	ΔΔ*G*_FOLD_ (kcal/mol)	Secondary structure change and accuracy of prediction (%)	ΔΔ*G*_BIND_ (kcal/mol)	*K*_A_ (nM)
1	E211A	36	66	− 0.04	Change, 0% sheet → sheet	− 0.55	115.56
2	H331A	32	66	1.27	None, 100%, helix → helix	− 0.57	113.43
3	H331R	− 22	61	0.98	None, 100%, helix → helix	− 0.83	72.65
4	R340A	65	80	1.88	None, 100%, helix → helix	− 0.76	81.21
5	D377A	72	85	0.74	None, 100%, coil → coil	− 0.07	261.56
6	D379A	27	63	− 0.08	None, 100%, helix → helix	− 0.33	168.65
7	D380A	36	66	− 2.73	None, 100%, helix → helix	− 0.52	123.00
8	T421A	45	71	0.94	None, 100%, helix → helix	− 0.97	57.36
9	T421S	− 2	53	0.48	None, 100%, helix → helix	− 0.53	119.52
10	N425A	31	66	0.77	None, 100%, helix → helix	− 0.73	86.29
11	D503A	45	71	0.83	None, 100%, coil → coil	− 0.02	283.63

Changes in the distance between aa residues which play important roles in catalysis or ligand stabilization are generally associated with changes in the catalytic properties observed in single mutants (Table S4). For example, D379 is localized closer to N425 in WT than in the mutated variants. In addition, in D377A, the distance D379:OD1-N425:HD2 (6.11 Å) was nearly three times than that observed in WT (2.12 Å), suggesting that N425 does not stabilize D379 and that it has a strong negative impact on its catalytic properties. However, in the mutated forms, D379 is closer to the substrate (GGPS) and T421 than in the wild type [3]. Contrary to the mutants believed to demonstrate improved catalytic properties, no statistically significant differences were observed.

Therefore, the most efficient parameters in screening out the unfavorable mutants were the high threshold of SNAP2 scores and a ΔΔ*G*_FOLD_ value lower than—0.50 kcal/mol. The first test removed all 11 mutants, while the second removed 10 of these 11 (90.9%). The other parameters used in the screening were found to be less efficient at selecting unfavorable mutants.

### Double Mutants and Double Mutant Cycle Analysis

The selected 6 single mutants of *A. thaliana ent*-CPS were used to build 15 double mutants. The obtained mutants are analysed in the same way as the single mutants, although the SNAP2 score test was omitted. A group of seven double mutants passing all quality tests was obtained (Table 3). All demonstrated greater affinity to the ligand GGPS, which varied between 77.33 nM (D377L/S597W) and 116.14 nM.(T114F/S597W) (Table 3). In addition, all seven double mutants induced minor negative changes to ΔΔ*G*_BIND_. Three of these seven double mutants (T114F/S597W, D336L/S597W and D336L/K778F) demonstrated moderately improved stability; however, another three (D377L/G422, D377L/S597W and D377L/K778F) displayed considerably greater enzyme stability, as measured by ΔΔ*G*_FOLD_ values (Table 3).

**Table 3:** Changes in secondary structure, ligand free folding energy, ligand free energy of binding and ligand affinity in fifteen double mutants of *A. thaliana ent*-CPS

Nr	Mutation	ΔΔ*G*_FOLD_ (kcal/mol)	Secondary structure change and accuracy of prediction (%)	ΔΔ*G*_BIND_ (kcal/mol)	*K*_A_ (nM)
1	T114F/D336L	1.83	None, 100%	− 0.64	97.61
2	**T114F/D377L**	− 0.89	None, 100%	− 0.60	106.56
3	T114F/G422L	− 2.87	None, 100%	− 0.18	216.51
4	**T114F/S597W**	− 1.62	None, 100%	− 0.55	116.14
5	T114F/K778F	0.30	None, 100%	− 0.02	282.68
6	D336L/D377L	− 1.94	None, 100%	− 0.48	129.39
7	D336L/G422L	− 1.93	None, 100%	− 0.10	249.07
8	**D336L/S597W**	− 1.84	None, 100%	− 0.74	84.56
9	**D336L/K778F**	− 1.67	None, 100%	− 0.79	77.46
10	**D377L/G422L**	− 3.24	None, 100%	− 0.72	87.03
11	**D377L/S597W**	− 2.43	None, 100%	− 0.79	77.33
12	**D377L/K778F**	− 2.43	None, 100%	− 0.79	78.12
13	G422L/S597 W	− 3.39	None, 100%	− 0.04	284.59
14	G422L/K778F	− 3.39	None, 100%	− 0.47	132.71
15	S597W/K778F	− 2.35	None, 100%	− 0.13	234.78

Seven selected double mutants that passed the presented quality test are marked in bold

Among the double mutants, the distances between aa residues important for catalysis are generally consistent with the biases observed in the single mutants that appear to demonstrate improved properties (Table S5), i.e., while D379 tends to be closer to the substrate and T421 in mutated forms, N425 is found to be further from D379 in mutants than in wild type (Table S5). None of the observed differences were statistically significant.

**Fig. 2:**
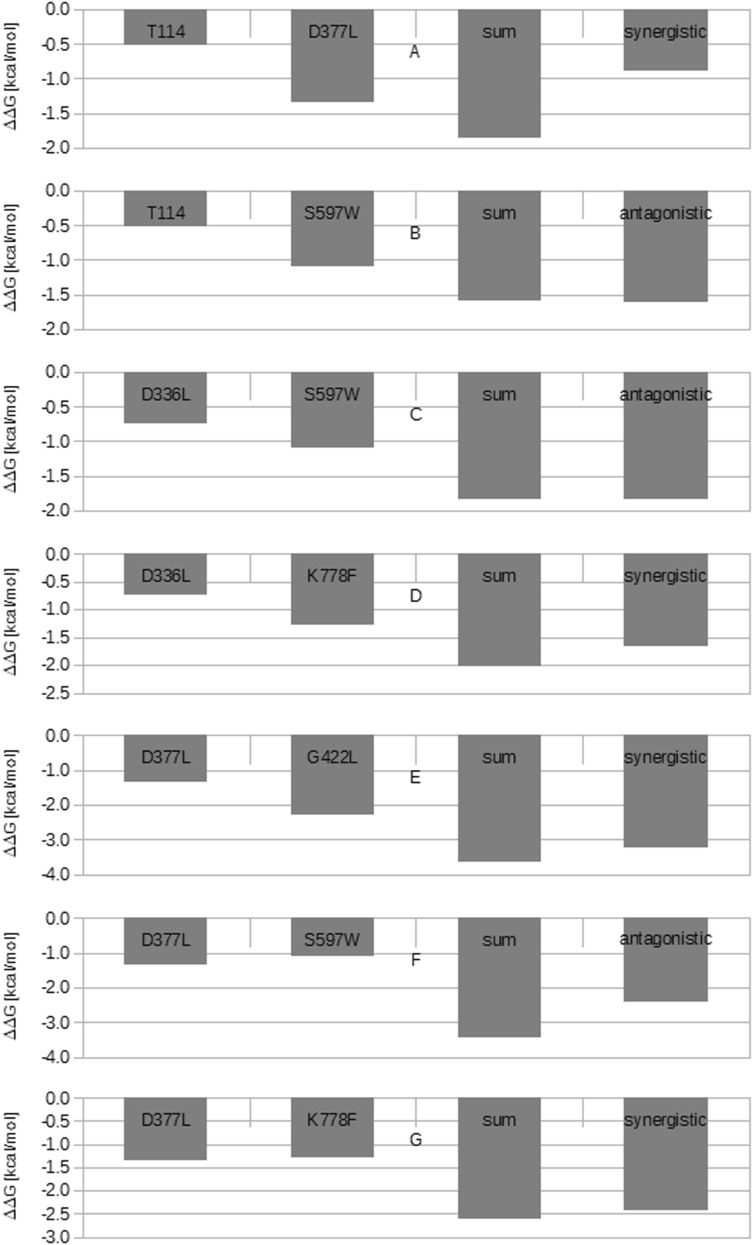
Double mutant cycle analysis of seven *A. thaliana ent*-CPS variants according to the ΔΔ_FOLD_ values

The presented seven double mutants were analysed by the double mutant cycle method, as described in Sect. 2. The tests were performed separately for ΔΔ*G*_FOLD_ and ΔΔ*G*_BIND_. The double mutant cycle analysis for ΔΔ*G*_FOLD_ indicated synergistic (T114F/D377L, D336L/K778F, D377L/G422L, D377L/K778F) and antagonistic (T114F/S597W, D336L/S597W, D377L/S597W) interactions between aa residues constituting the double mutant (Table 4, Fig. 2). The antagonistic effects within T114F/S597W and D336L/S597W were found to be very weak, representing only 0.02 and 0.01 kcal/mol, respectively. It is possible that both double mutants are in fact built from single mutants, demonstrating an additive form of interaction rather than an antagonistic one. The biological influence on protein stability exerted by each single mutant may be defined according to Mildvan [37]. The presence of an antagonistic interaction suggests that opposing structural effects exist within the same thermodynamic step, while that of a synergistic interaction implies the existence of anti-cooperative interactions between residues facilitating the same thermodynamic step. Finally, the presence of an additive effect indicates that the aa residues building the double mutant do not interact during the same thermodynamic step.

**Table 4:** Results of double mutant cycle analysis based on ΔΔ*G*_FOLD_

Nr	Mutation	ΔΔ*G*_FOLD_ of single mutant (kcal/mol)	Sum of ΔΔ*G*_FOLD_ of single mutants (kcal/mol)	ΔΔ*G*_FOLD_ of double mutant (kcal/mol)	Interaction type according to Mildvan et al. [37]
1	T114/D377L	− 0.51/− 1.34	− 1.85	− 0.89	Synergistic
2	T114F/S597W	− 0.51/− 1.09	− 1.60	− 1.62	Antagonistic^a^
3	D336L/S597W	− 0.74/− 1.09	− 1.83	− 1.84	Antagonistic^a^
4	D336L/K778F	− 0.74/− 1.28	− 2.02	− 1.67	Synergistic
5	D377L/G422L	− 1.34/− 2.31	− 3.65	− 3.24	Synergistic
6	D377L/S597W	− 1.34/− 1.09	− 3.43	− 2.43	Antagonistic
7	D377L/K778F	− 1.34/− 1.28	− 2.62	− 2.43	Synergistic

The superscript letter (a) indicates a very weak antagonistic interaction that could in fact be the additive one

**Fig. 3:**
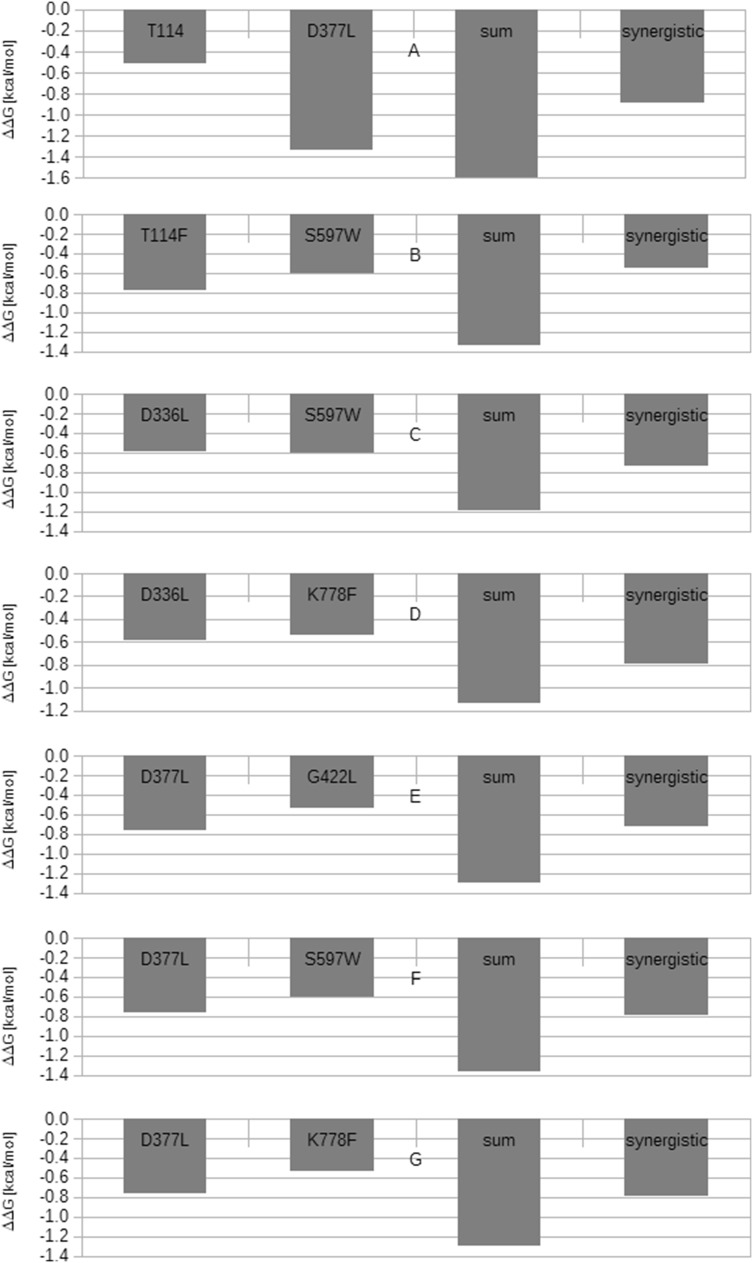
Double mutant cycle analysis of seven *A. thaliana ent*-CPS variants according to the ΔΔ_BIND_ values

However, all seven combinations have been found to demonstrate synergistic effects on ΔΔG_BIND_, suggesting that the components do not in fact interact, and facilitate the same non-rate-limiting step [37] (Table 5, Fig. 3).

**Table 5:** Results of double mutant cycle analysis based on ΔΔ*G*_BIND_

Nr	Mutation	ΔΔ*G*_BIND_ of single mutant (kcal/mol)	Sum of ΔΔ*G*_BIND_ of single mutants (kcal/mol)	ΔΔ*G*_BIND_ of double mutant (kcal/mol)	Interaction type according to Mildvan et al. [37]
1	T114/D377L	− 0.77/− 0.76	− 1.53	− 0.60	Synergistic
2	T114F/S597W	− 0.77/− 0.60	− 1.33	− 0.55	Synergistic
3	D336L/S597W	− 0.59/− 0.60	− 1.19	− 0.74	Synergistic
4	D336L/K778F	− 0.59/− 0.54	− 1.13	− 0.79	Synergistic
5	D377L/G422L	− 0.76/− 0.54	− 1.30	− 0.72	Synergistic
6	D377L/S597W	− 0.76/− 0.60	− 1.36	− 0.79	Synergistic
7	D377L/K778F	− 0.76/− 0.54	− 1.30	− 0.79	Synergistic

## Discussion

The SNAP2 tool allowed 455 non-neutral *A. thaliana ent*-CPS variants to be selected from the entire population of 15,257 mutants; of these, 14 mutants with potentially stabilizing properties were identified using ΔΔ*G*_FOLD_. Among these, eight (57.14%) displayed moderate or strong stabilization functions. The importance of quantitative ΔΔ*G*_FOLD_ stabilization measurement depends on the properties of FoldX software, which more accurately predicts destabilizing mutations than stabilizing ones [31]. A correlation of 0.81 was calculated between predicted and experimental values for more than 1000 point mutations with an accuracy of 0.46 kcal/mol [29]. Recent analyses have found the FoldX standard deviation (SD) of predicted real positives and real negatives to be 1.0–1.78 kcal/mol; hence, mutants with highly stabilizing properties are also highly likely to demonstrate these stabilization properties in vivo [31].

Further analysis indicated that five variants, i.e., two minor (H391L, H391M), two moderate (H793L, H793M) and one strong (H793F), showed changes in secondary structure that could negatively affect the catalytic properties of the enzyme. Three of the five mutants (H793F, H793L, H793M) are localized on the C-terminal segment of the protein; this region may be less stabilized by interactions with other aa residues, making the mutant forms more vulnerable to inconvenient structural changes.

Of the nine variants selected on the basis of ΔΔ*G*_FOLD_ and the lack of any change in secondary structure, six also displayed minor, negative changes in ΔΔ*G*_BIND_, suggesting that the mutants demonstrated stronger ligand binding. Mutant forms were also found to demonstrate some tendencies regarding the distances between the ligand and the aa residues playing a crucial role in a catalytic reaction: D379 seems to interact more closely with the ligand in the mutated forms than in the WT, and T421 may stabilize D379 more strongly in six mutant variants, while N425 may have a weaker influence on D379 in mutants than in the WT.

The relationship between the length of the hydrogen bond and its energy was characterized by Jeffrey [43], who notes that hydrogen bonds with donor–acceptor distances of 2.2–2.5 Å are strong, 2.5–3.2 Å are moderate, and 3.2–4.0 Å are weak. The 4.02 Å distance between D379:OD2 and GGPS-C15 therefore suggests a weak interaction, while the much shorter length (2.12 Å) of D379:OD1-N425:HD2 bond indicates a strong interaction. In addition, assuming that the 5.53 Å D379:OD2-T421:HG1 bond is mediated by a water molecule, its strength could be categorized as moderate [3, 43].

The tools used in the study were successfully validated on a group of 11 available *A. thaliana ent*-CPS mutants of decreased catalytic activity. This procedure efficiently eliminated all variants with unfavorable catalytic properties.

The study went on to examine the properties of 15 double mutants constructed from 6 single mutants, which were found to offer both improved stability and greater ligand affinity. Following screening with the same tools used for the single mutants, a group of seven mutants were found to display greater stabilization and ligand affinity. Similarly to the single mutants, all double mutants showed minor changes in ΔΔ_BIND_ and demonstrated a synergistic effect on ΔΔ*G*_BIND_, indicating that the non-interacting residues facilitate the same catalytic, non-rate-limiting step [37].

In addition, the double mutant cycle analysis for ΔΔ*G*_FOLD_ indicated the presence of antagonistic (T114F/S597W, D336L/S597W, D377L/S597W) interactions between aa residues constituting the double mutant. As the antagonistic effect was very weak, it is possible that both double mutants (T114F/S597W, D336L/S597W) are in fact formed from single mutants, suggesting an additive effect takes place rather than an antagonistic one. The presence of an antagonistic effect indicates that the two single mutants forming the double mutant exert opposing activities on the same thermodynamic step; however, the fact that the antagonistic effect is very weak suggests that the interaction could be additive, and that the single mutants could not interact in the stabilization process [37].

The next four double mutants (T114F/D377L, D336L/K778F, D377L/G422L, D377L/K778F) showed a synergistic effect on protein ΔΔ*G*_FOLD_, suggesting the presence of anti-cooperatively interacting residues.

Besides the double mutant cycle analysis, the potential functional interactions acting between aa residues located at a distance could be explained by the recently introduced concept of protein sectors. Halabi [44] propose that proteins are organized into groups of aa residues that are structurally and functionally connected but separated in terms of primary structure. These groups, known as protein sectors, are built from coevolving aa residues [44].

The sectors are built around an active site and indicate structural and biochemical independence [45]. Therefore, the mutation of aa residues in a particular sector of *A. thaliana ent*-CPS may be of greatest importance to protein function or stability. However, no sector or statistical coupling analysis has yet been performed on *ent*-CPS or (+)-CPS enzymes [46, 47].

Future research should attempt to validate the properties of the obtained single and double mutants, particularly D336L and the four double mutants containing this substitution. D336 is part of the Grotthus diffusion-based channel participating in the reprotonation of D379 in the catalytic cycle [3]. The exchange of the acidic, polar side chain of D336 into a hydrophobic L336 could strongly decrease the efficiency of this D379 reprotonation, potentially leading to diminished enzyme activity. Experimentally verified single and double mutants of *A. thaliana ent*-CPS could be used to prepare transgenic plants with greater *ent*-copalyl diphosphate and gibberellin productivity that could be used to study hormone-dependent gene expression. In addition, the improved endogenous supply of gibberellins could efficiently stimulate the expression of hormone-regulated genes, boosting the biosynthesis of valuable secondary metabolites, such as tanshinones in red sage.

## Conclusion

The presented methodology allowed 9 mutants with improved ΔΔ*G*_FOLD_ to be selected from the 15,257 members of the *A. thaliana ent*-CPS saturation mutagenesis library. The selected nine forms of *A. thaliana ent*-CPS included six mutants that combine improved stability (ΔΔ*G*_FOLD_) with increased affinity (ΔΔ*G*_BIND_) to the GGPS ligand. Seven double mutants built from the six single mutants demonstrated a synergistic effect on ΔΔ*G*_BIND_, suggesting a lack of interaction between the analysed aa residues. A ΔΔ*G*_FOLD_-based analysis of protein stability found three double mutants to display an antagonistic effect and four to show a synergistic mode of interaction. Our findings increase the number of known *A. thaliana ent*-CPS single and double mutants displaying potentially improved stability and ligand affinity. Following experimental verification, these forms could be used to further investigate gibberellin metabolism regulation in plants. These findings further clarify the interactions between selected *A. thaliana ent*-CPS aa residues facilitating ligand affinity and protein stability.

## Electronic supplementary material

Below is the link to the electronic supplementary material.

Supplementary material 1 (DOCX 41 kb)

## Abbreviations

*ent*-CPS: *Ent*-copalyl diphosphate synthase
GGPS: Geranylgeranyldiphosphate
SNAP 2: Screening for non-acceptable polymorphisms 2
