# Supplementary material for: Computer-Aided Saturation Mutagenesis of Arabidopsis thaliana Ent-Copalyl Diphosphate Synthase
Source: Interdiscip Sci. 2019 Jul 15;12(1):32–43. doi: 10.1007/s12539-019-00342-x (PMC7007437; doi:10.1007/s12539-019-00342-x)
Supplement: Supplementary file 1 — Supplementary material 1 (DOCX 41 kb) [file 12539_2019_342_MOESM1_ESM.docx]

**Supplementary material**

**Tables**

Table S1 A group of 455 variants of *A. thaliana* *ent*-CPS obtained from a saturation mutagenesis library containing 15257 mutants produced by SNAP2 tool. Selected 455 variants indicated threshold values of effect score higher than 85 and a probability of at least 91%. The ΔΔG_FOLD_ values were calculated for each mutant.

| **Nr** | **Mutation** | **Effect score [-100;100]** | **Probability [%]** | **ΔΔG_FOLD_ [kcal/mol]** |
| --- | --- | --- | --- | --- |
| 1 | G111R | 87 | 91% | 19.99 |
| 2 | I113K | 85 | 91% | 5.06 |
| 3 | T114D | 87 | 91% | 0.72 |
| 4 | T114E | 89 | 91% | 0.35 |
| 5 | T114F | 88 | 91% | -0.51 |
| 6 | T114H | 86 | 91% | 0.57 |
| 7 | T114K | 91 | 95% | - 0.10 |
| 8 | T114L | 86 | 91% | -0.36 |
| 9 | T114R | 91 | 95% | 0.05 |
| 10 | T114W | 90 | 95% | - 0.05 |
| 11 | S116D | 86 | 91% | 16.74 |
| 12 | S116F | 85 | 91% | 21.76 |
| 13 | S116K | 87 | 91% | 16.34 |
| 14 | S116P | 88 | 91% | 9.16 |
| 15 | S116R | 88 | 91% | 20.37 |
| 16 | S116W | 89 | 91% | 39.96 |
| 17 | S116Y | 85 | 91% | 24.36 |
| 18 | Y118A | 97 | 95% | 5.16 |
| 19 | Y118C | 95 | 95% | 5. 52 |
| 20 | Y118D | 98 | 95% | 7. 53 |
| 21 | Y118E | 98 | 95% | 6.40 |
| 22 | Y118F | 92 | 95% | 0.77 |
| 23 | Y118G | 98 | 95% | 6.99 |
| 24 | Y118H | 97 | 95% | 3.08 |
| 25 | Y118I | 95 | 95% | 5. 55 |
| 26 | Y118L | 97 | 95% | 1.83 |
| 27 | Y118K | 98 | 95% | 3. 55 |
| 28 | Y118M | 97 | 95% | 2.12 |
| 29 | Y118N | 98 | 95% | 5.05 |
| 30 | Y118P | 99 | 95% | 10.87 |
| 31 | Y118R | 98 | 95% | 4.40 |
| 32 | Y118S | 98 | 95% | 6.24 |
| 33 | Y118T | 98 | 95% | 5.75 |
| 34 | Y118V | 96 | 95% | 4.72 |
| 35 | Y118W | 95 | 95% | 3.79 |
| 36 | D119A | 88 | 91% | 2.83 |
| 37 | D119C | 96 | 91% | 2.89 |
| 38 | D119F | 94 | 95% | 10.51 |
| 39 | D119G | 95 | 95% | 3.92 |
| 40 | D119H | 93 | 95% | 7.87 |
| 41 | D119I | 94 | 95% | 5.42 |
| 42 | D119K | 95 | 95% | 17.49 |
| 43 | D119L | 95 | 95% | 5.10 |
| 44 | D119M | 93 | 95% | 4.29 |
| 45 | D119N | 89 | 91% | 3.45 |
| 46 | D119P | 95 | 95% | 5.13 |
| 47 | D119Q | 91 | 95% | 7.07 |
| 48 | D119R | 95 | 95% | 21.01 |
| 49 | D119T | 89 | 91% | 6.10 |
| 50 | D119V | 93 | 95% | 3.64 |
| 51 | D119W | 96 | 95% | 22.98 |
| 52 | D119Y | 94 | 95% | 15.25 |
| 53 | T120D | 86 | 91% | 10.90 |
| 54 | T120E | 90 | 95% | 10.90 |
| 55 | T120F | 89 | 91% | 26.83 |
| 56 | T120H | 86 | 91% | 16.36 |
| 57 | T120I | 85 | 91% | 4.83 |
| 58 | T120K | 91 | 95% | 12.89 |
| 59 | T120L | 87 | 91% | 3.48 |
| 60 | T120P | 86 | 91% | 7.77 |
| 61 | T120R | 91 | 95% | 19.63 |
| 62 | T120W | 91 | 95% | 41.77 |
| 63 | T120Y | 87 | 91% | 24.25 |
| 64 | W122D | 86 | 91% | 5.19 |
| 65 | W122P | 91 | 95% | 6.39 |
| 66 | F135D | 94 | 95% | 0. 66 |
| 67 | F135E | 91 | 95% | 1. 62 |
| 68 | F135G | 91 | 95% | 5.01 |
| 69 | F135K | 91 | 95% | 3.01 |
| 70 | F135N | 90 | 95% | 1.86 |
| 71 | F135P | 94 | 95% | 5. 55 |
| 72 | F135Q | 88 | 91% | 3.67 |
| 73 | F135R | 89 | 91% | 4.18 |
| 74 | F135S | 88 | 91% | 3.25 |
| 75 | F135T | 87 | 91% | 3.35 |
| 76 | W141D | 91 | 95% | 5.03 |
| 77 | W141E | 86 | 91% | 3.29 |
| 78 | W141G | 89 | 91% | 4.51 |
| 79 | W141K | 90 | 95% | 2.85 |
| 80 | W141L | 85 | 91% | 1.54 |
| 81 | W141N | 86 | 91% | 3.60 |
| 82 | W141P | 95 | 95% | 7.65 |
| 83 | W141S | 86 | 91% | 3.84 |
| 84 | W141T | 88 | 91% | 4.15 |
| 85 | G150D | 93 | 95% | 13.07 |
| 86 | G150E | 94 | 95% | 13.49 |
| 87 | G150F | 91 | 95% | 14.90 |
| 88 | G150H | 88 | 91% | 21.55 |
| 89 | G150I | 90 | 95% | 14.42 |
| 90 | G150K | 95 | 95% | 12.82 |
| 91 | G150L | 90 | 95% | 12.47 |
| 92 | G150M | 90 | 95% | 9.78 |
| 93 | G150P | 94 | 95% | 8.13 |
| 94 | G150Q | 88 | 91% | 11.27 |
| 95 | G150R | 90 | 95% | 10.47 |
| 96 | G150T | 89 | 91% | 11.09 |
| 97 | G150V | 87 | 91% | 11.78 |
| 98 | G150W | 93 | 95% | 19.77 |
| 99 | G150Y | 90 | 95% | 14.98 |
| 100 | W152A | 92 | 95% | 4.40 |
| 101 | W152D | 97 | 95% | 7.84 |
| 102 | W152E | 95 | 95% | 5.42 |
| 103 | W152G | 95 | 95% | 5.39 |
| 104 | W152H | 94 | 95% | 3.72 |
| 105 | W152I | 89 | 91% | 3.17 |
| 106 | W152K | 96 | 95% | 3.44 |
| 107 | W152L | 91 | 95% | 3.17 |
| 108 | W152M | 89 | 91% | 2.68 |
| 109 | W152N | 95 | 95% | 3.88 |
| 110 | W152P | 97 | 95% | 5.13 |
| 111 | W152Q | 92 | 95% | 4.71 |
| 112 | W152R | 95 | 95% | 3.72 |
| 113 | W152S | 94 | 95% | 4.11 |
| 114 | W152T | 94 | 95% | 4.07 |
| 115 | W152V | 91 | 95% | 2.30 |
| 116 | G153E | 86 | 91% | 20.78 |
| 117 | G153F | 86 | 91% | 28.74 |
| 118 | G153K | 90 | 95% | 21.27 |
| 119 | G153L | 85 | 91% | 25.04 |
| 120 | G153P | 87 | 91% | 14.55 |
| 121 | G153R | 89 | 91% | 25.87 |
| 122 | G153W | 89 | 91% | 49.22 |
| 123 | G153Y | 86 | 91% | 29.58 |
| 124 | D162K | 89 | 91% | 7.10 |
| 125 | D162P | 85 | 91% | 3.27 |
| 126 | D162R | 86 | 91% | 10.83 |
| 127 | D162W | 87 | 91% | 12.71 |
| 128 | R163D | 91 | 95% | 7.90 |
| 129 | R163E | 88 | 91% | 7.25 |
| 130 | R163G | 87 | 91% | 4.37 |
| 131 | R163P | 91 | 95% | 5.81 |
| 132 | R163W | 91 | 95% | 8.44 |
| 133 | T167F | 85 | 91% | 22.40 |
| 134 | T167K | 85 | 91% | 7.70 |
| 135 | T167W | 88 | 91% | 33.47 |
| 136 | A169K | 85 | 91% | 6.00 |
| 137 | A169W | 85 | 91% | 13.66 |
| 138 | C170W | 87 | 91% | 29.62 |
| 139 | W177D | 88 | 91% | 4.47 |
| 140 | W177G | 85 | 91% | 3.23 |
| 141 | G187E | 86 | 91% | 17.71 |
| 142 | G187F | 85 | 91% | 41.68 |
| 143 | G187K | 90 | 95% | 18.99 |
| 144 | G187P | 86 | 91% | 7.29 |
| 145 | G209H | 86 | 91% | 2.18 |
| 146 | G209K | 85 | 91% | 2.13 |
| 147 | G209P | 87 | 91% | 5.66 |
| 148 | F210D | 91 | 95% | 5.34 |
| 149 | F210E | 86 | 91% | 5.36 |
| 150 | F210K | 88 | 91% | 3.75 |
| 151 | F210P | 90 | 95% | 5.98 |
| 152 | F210R | 86 | 91% | 5.78 |
| 153 | F214D | 92 | 95% | 6.44 |
| 154 | F214E | 88 | 91% | 6.03 |
| 155 | F214H | 85 | 91% | 4.22 |
| 156 | F214K | 91 | 95% | 5.44 |
| 157 | F214N | 87 | 91% | 5.85 |
| 158 | F214P | 92 | 95% | 10.84 |
| 159 | F214Q | 85 | 91% | 4.59 |
| 160 | F214R | 89 | 91% | 7.81 |
| 161 | P215K | 85 | 91% | 8.53 |
| 162 | P215N | 87 | 91% | 4.14 |
| 163 | E266R | 90 | 95% | 10.97 |
| 164 | E266W | 85 | 91% | 17.64 |
| 165 | G267D | 85 | 91% | 4.07 |
| 166 | G267K | 90 | 95% | 2.49 |
| 167 | G267R | 89 | 91% | 3.77 |
| 168 | W273D | 85 | 91% | 4.65 |
| 169 | W273P | 90 | 95% | 1. 61 |
| 170 | F286D | 86 | 91% | 5.12 |
| 171 | S291D | 88 | 91% | 14.13 |
| 172 | S291E | 86 | 91% | 14.14 |
| 173 | S291P | 87 | 91% | 4.53 |
| 174 | S291R | 86 | 91% | 23.19 |
| 175 | S291W | 88 | 91% | 28.73 |
| 176 | D336F | 85 | 91% | 0.81 |
| 177 | D336H | 85 | 91% | 1.87 |
| 178 | D336K | 91 | 95% | 2.37 |
| 179 | D336L | 87 | 91% | -0.74 |
| 180 | D336P | 89 | 91% | 3.20 |
| 181 | D336R | 91 | 95% | 4.07 |
| 182 | D336V | 85 | 91% | -0.08 |
| 183 | D336W | 90 | 95% | 1.78 |
| 184 | D336Y | 86 | 91% | 0.21 |
| 185 | R340D | 88 | 91% | 4.08 |
| 186 | R340P | 86 | 91% | 4.90 |
| 187 | R340W | 85 | 91% | -0.38 |
| 188 | L341D | 88 | 91% | 4.77 |
| 189 | L341K | 85 | 91% | 3.68 |
| 190 | L341P | 87 | 91% | 6.62 |
| 191 | L341R | 85 | 91% | 5.09 |
| 192 | G342E | 87 | 91% | 16.24 |
| 193 | G342K | 88 | 91% | 17.89 |
| 194 | G342M | 85 | 91% | 14.72 |
| 195 | G342P | 85 | 91% | 18.31 |
| 196 | G342W | 86 | 91% | 41.31 |
| 197 | G342Y | 87 | 91% | 38.41 |
| 198 | Y346P | 88 | 91% | 3.52 |
| 199 | I351P | 85 | 91% | 5.74 |
| 200 | L355P | 87 | 91% | 5.85 |
| 201 | Y357P | 86 | 91% | 2.87 |
| 202 | Y361P | 87 | 91% | 5.96 |
| 203 | W362D | 87 | 91% | 7.71 |
| 204 | W362K | 85 | 91% | 4.35 |
| 205 | W362P | 91 | 95% | 9.84 |
| 206 | W369P | 88 | 91% | 0.96 |
| 207 | D377F | 86 | 91% | 0.18 |
| 208 | D377I | 88 | 91% | -0.03 |
| 209 | D377L | 88 | 91% | -1.34 |
| 210 | D377K | 87 | 91% | -0.72 |
| 211 | D377M | 86 | 91% | -0.38 |
| 212 | D377P | 92 | 95% | 5.44 |
| 213 | D377R | 89 | 91% | 0.24 |
| 214 | D377W | 91 | 95% | 0.14 |
| 215 | D377Y | 90 | 95% | - 0.34 |
| 216 | R386D | 87 | 91% | 2.82 |
| 217 | R389F | 87 | 91% | 10.85 |
| 218 | R389G | 86 | 91% | 6.65 |
| 219 | R389P | 92 | 95% | 8.71 |
| 220 | R389W | 91 | 95% | 7.78 |
| 221 | H391D | 93 | 95% | 2.22 |
| 222 | H391I | 89 | 91% | 1.28 |
| 223 | H391K | 89 | 91% | 0.66 |
| 224 | H391L | 87 | 91% | -0.83 |
| 225 | H391M | 88 | 91% | -0.67 |
| 226 | H391P | 95 | 95% | 3.87 |
| 227 | H391R | 92 | 95% | 1.07 |
| 228 | H391T | 89 | 91% | 1.51 |
| 229 | H391W | 93 | 95% | 1.90 |
| 230 | G392E | 87 | 91% | 3.55 |
| 231 | G392F | 85 | 91% | 2.18 |
| 232 | G392K | 90 | 95% | 3.27 |
| 233 | G392L | 85 | 91% | 3.10 |
| 234 | G392P | 88 | 91% | 5.68 |
| 235 | G392W | 90 | 95% | 2.81 |
| 236 | Y393D | 88 | 91% | 4.07 |
| 237 | Y393E | 87 | 91% | 2.55 |
| 238 | Y393K | 88 | 91% | 3.06 |
| 239 | D398L | 85 | 91% | 0.46 |
| 240 | Y393N | 86 | 91% | 1.13 |
| 241 | D398H | 86 | 91% | 0.99 |
| 242 | D398K | 87 | 91% | 0.43 |
| 243 | D398P | 89 | 91% | 3.70 |
| 244 | D398R | 85 | 91% | 0.20 |
| 245 | D398W | 89 | 91% | 1.46 |
| 246 | V420H | 85 | 91% | 5.03 |
| 247 | V420K | 87 | 91% | 2.52 |
| 248 | V420W | 85 | 91% | 6.30 |
| 249 | G422E | 89 | 91% | 0.10 |
| 250 | G422L | 89 | 91% | -2.31 |
| 251 | G422M | 87 | 91% | -2.88 |
| 252 | G422N | 88 | 91% | 0.04 |
| 253 | M423K | 85 | 91% | 2.13 |
| 254 | M423P | 87 | 91% | 4.17 |
| 255 | M423R | 85 | 91% | 4.35 |
| 256 | F424D | 86 | 91% | 5.30 |
| 257 | F424H | 90 | 95% | 3.27 |
| 258 | F424I | 90 | 95% | 3.41 |
| 259 | F424K | 95 | 95% | 2.46 |
| 260 | F424Q | 91 | 95% | 3.77 |
| 261 | F424R | 94 | 95% | 4.42 |
| 262 | F424W | 94 | 95% | 2.42 |
| 263 | F424Y | 93 | 95% | 1.73 |
| 264 | E437A | 88 | 91% | 3.93 |
| 265 | E437C | 88 | 91% | 3.74 |
| 266 | E437D | 86 | 91% | 2.96 |
| 267 | E437F | 95 | 95% | 2.91 |
| 268 | E437G | 93 | 95% | 5.02 |
| 269 | E437H | 89 | 91% | 3.49 |
| 270 | E437I | 94 | 95% | 4.35 |
| 271 | E437L | 94 | 95% | 3.05 |
| 272 | E437K | 94 | 95% | 3.58 |
| 273 | E437M | 93 | 95% | 4.01 |
| 274 | E437P | 97 | 95% | 6.80 |
| 275 | E437Q | 94 | 95% | 3.80 |
| 276 | E437R | 95 | 95% | 4.38 |
| 277 | E437S | 89 | 91% | 4.44 |
| 278 | E437T | 90 | 95% | 4.45 |
| 279 | E437V | 94 | 95% | 4.00 |
| 280 | E437W | 96 | 95% | 3.77 |
| 281 | E437Y | 94 | 95% | 3.59 |
| 282 | I439D | 93 | 95% | 2.08 |
| 283 | I439E | 88 | 91% | 1.30 |
| 284 | I439F | 85 | 91% | 0.92 |
| 285 | I439G | 93 | 95% | 1.48 |
| 286 | I439H | 90 | 95% | 1.72 |
| 287 | I439K | 92 | 95% | 0.56 |
| 288 | I439N | 92 | 95% | 0.46 |
| 289 | I439P | 94 | 95% | 0.19 |
| 290 | I439Q | 87 | 91% | 0.78 |
| 291 | I439R | 88 | 91% | 0.61 |
| 292 | I439S | 89 | 91% | 1.27 |
| 293 | I439T | 87 | 91% | 0.65 |
| 294 | I439W | 91 | 95% | 1.34 |
| 295 | I439Y | 89 | 91% | 0.89 |
| 296 | L440D | 93 | 95% | 4.80 |
| 297 | L440E | 85 | 91% | 4.16 |
| 298 | L440G | 92 | 95% | 4.56 |
| 299 | L440H | 88 | 91% | 3.73 |
| 300 | L440K | 92 | 95% | 5.07 |
| 301 | L440N | 91 | 95% | 3.90 |
| 302 | L440P | 94 | 95% | 2.33 |
| 303 | L440R | 91 | 95% | 5.91 |
| 304 | L440S | 86 | 91% | 3.28 |
| 305 | L440T | 88 | 91% | 3.24 |
| 306 | L440W | 88 | 91% | 14.92 |
| 307 | L440Y | 85 | 91% | 4.03 |
| 308 | F446P | 86 | 91% | 5.04 |
| 309 | D462K | 85 | 91% | 9.34 |
| 310 | W464P | 85 | 91% | 4.43 |
| 311 | L478D | 86 | 91% | 5.85 |
| 312 | L478K | 86 | 91% | 4.23 |
| 313 | L478P | 87 | 91% | 4.83 |
| 314 | W482K | 86 | 91% | 3.66 |
| 315 | W482P | 90 | 95% | 2.69 |
| 316 | Y483D | 85 | 91% | 4.80 |
| 317 | Y483P | 87 | 91% | 1.23 |
| 318 | R488E | 92 | 95% | 1.52 |
| 319 | R488F | 91 | 95% | 1.77 |
| 320 | R488G | 92 | 95% | 2.09 |
| 321 | R488I | 86 | 91% | 2.89 |
| 322 | R488N | 89 | 91% | 0.63 |
| 323 | R488P | 91 | 95% | 2.20 |
| 324 | R488S | 87 | 91% | 2.22 |
| 325 | R488V | 87 | 91% | 2.72 |
| 326 | R488W | 92 | 95% | 11.32 |
| 327 | R488Y | 89 | 91% | 4.09 |
| 328 | Y494P | 86 | 91% | 2.89 |
| 329 | W505D | 86 | 91% | 7.14 |
| 330 | W505P | 90 | 95% | 8.06 |
| 331 | K508D | 85 | 91% | 4.31 |
| 332 | R512E | 89 | 91% | 3.67 |
| 333 | R512F | 87 | 91% | -0.33 |
| 334 | R512G | 88 | 91% | 2.11 |
| 335 | R512P | 91 | 95% | 1.90 |
| 336 | R512Y | 86 | 91% | -0.40 |
| 337 | M513P | 87 | 91% | 4.64 |
| 338 | N518W | 86 | 91% | 0.25 |
| 339 | Y521K | 85 | 91% | 2.01 |
| 340 | Y521P | 87 | 91% | 1.13 |
| 341 | D528W | 86 | 91% | 2.66 |
| 342 | Y529D | 90 | 95% | 3.79 |
| 343 | Y529E | 91 | 95% | 3.23 |
| 344 | Y529G | 87 | 91% | 3.36 |
| 345 | Y529K | 91 | 95% | 3.04 |
| 346 | Y529N | 86 | 91% | 3.96 |
| 347 | Y529P | 94 | 95% | 5.37 |
| 348 | Y529Q | 88 | 91% | 2.33 |
| 349 | Y529R | 90 | 95% | 3.39 |
| 350 | E539K | 88 | 91% | 2.89 |
| 351 | E539P | 90 | 95% | 5.40 |
| 352 | E539R | 89 | 91% | 3.41 |
| 353 | E539W | 87 | 91% | 6.18 |
| 354 | W546A | 86 | 91% | 5.85 |
| 355 | W555P | 85 | 91% | 2.71 |
| 356 | L562D | 85 | 91% | 4.11 |
| 357 | Y566K | 85 | 91% | 3.55 |
| 358 | Y566P | 87 | 91% | 10.89 |
| 359 | Y567D | 85 | 91% | 5.39 |
| 360 | Y567K | 88 | 91% | 4.46 |
| 361 | Y567P | 92 | 95% | 8.26 |
| 362 | Y567R | 86 | 91% | 4.85 |
| 363 | A571D | 90 | 95% | 7.27 |
| 364 | A571E | 90 | 95% | 10.19 |
| 365 | A571F | 90 | 95% | 31.63 |
| 366 | A571H | 90 | 95% | 22.20 |
| 367 | A571K | 92 | 95% | 14.24 |
| 368 | A571L | 89 | 91% | 6.75 |
| 369 | A571P | 88 | 91% | 6.87 |
| 370 | A571Q | 86 | 91% | 9.67 |
| 371 | A571R | 91 | 95% | 15.46 |
| 372 | A571W | 93 | 95% | 37.85 |
| 373 | A571Y | 89 | 91% | 35.78 |
| 374 | R582D | 95 | 95% | 9.28 |
| 375 | R582E | 91 | 95% | 8.69 |
| 376 | R582F | 90 | 95% | 7.11 |
| 377 | R582G | 89 | 91% | 8.21 |
| 378 | R582L | 85 | 91% | 5.04 |
| 379 | R582N | 85 | 91% | 7.39 |
| 380 | R582P | 94 | 95% | 13.42 |
| 381 | R582V | 85 | 91% | 8.27 |
| 382 | R582W | 94 | 95% | 15.45 |
| 383 | R582Y | 89 | 91% | 11.55 |
| 384 | W585P | 88 | 91% | 10.27 |
| 385 | A586W | 86 | 91% | 41.14 |
| 386 | K587W | 87 | 91% | 2.31 |
| 387 | L591D | 85 | 91% | 5.49 |
| 388 | L591P | 85 | 91% | 8.88 |
| 389 | S597P | 89 | 91% | 1.46 |
| 390 | S597W | 89 | 91% | -1.09 |
| 391 | F599D | 86 | 91% | 4.93 |
| 392 | F599P | 85 | 91% | 4.81 |
| 393 | L653P | 85 | 91% | 7.36 |
| 394 | W679E | 86 | 91% | 7.45 |
| 395 | W679H | 85 | 91% | 4.56 |
| 396 | W679N | 85 | 91% | 6.17 |
| 397 | W679P | 93 | 95% | 9.10 |
| 398 | W679R | 88 | 91% | 6.55 |
| 399 | W679T | 85 | 91% | 5.72 |
| 400 | L694D | 87 | 91% | 4.99 |
| 401 | L694K | 85 | 91% | 6.61 |
| 402 | L694G | 86 | 91% | 4.11 |
| 403 | L694R | 85 | 91% | 12.53 |
| 404 | L719D | 86 | 91% | 5.60 |
| 405 | L719P | 87 | 91% | 7.36 |
| 406 | N724P | 85 | 91% | 1.65 |
| 407 | N724W | 89 | 91% | 1.64 |
| 408 | R725F | 85 | 91% | 1.49 |
| 409 | M752D | 85 | 91% | 4.89 |
| 410 | M752P | 87 | 91% | 7.03 |
| 411 | K778D | 91 | 95% | 4.46 |
| 412 | K778E | 90 | 95% | 2.52 |
| 413 | K778F | 88 | 91% | -1.28 |
| 414 | K778P | 91 | 95% | 4.95 |
| 415 | K778W | 92 | 95% | - 0.90 |
| 416 | K778Y | 87 | 91% | -0.70 |
| 417 | F780D | 88 | 91% | 3.42 |
| 418 | F780K | 85 | 91% | 1.33 |
| 419 | Y781E | 90 | 95% | 5.17 |
| 420 | Y781G | 89 | 91% | 5.34 |
| 421 | Y781K | 91 | 95% | 3.50 |
| 422 | Y781P | 94 | 95% | 7.53 |
| 423 | Y781R | 90 | 95% | 3.97 |
| 424 | Y781S | 88 | 91% | 5.13 |
| 425 | Y781T | 88 | 91% | 4.68 |
| 426 | Y781W | 86 | 91% | -0.37 |
| 427 | Y782A | 87 | 91% | 3.38 |
| 428 | Y782D | 95 | 95% | 5.51 |
| 429 | Y782E | 95 | 95% | 3.41 |
| 430 | Y782G | 94 | 95% | 5.20 |
| 431 | Y782H | 89 | 91% | 1.50 |
| 432 | Y782I | 89 | 91% | 3.82 |
| 433 | Y782K | 95 | 95% | 2.73 |
| 434 | Y782L | 91 | 95% | 2.51 |
| 435 | Y782M | 90 | 95% | 1.26 |
| 436 | Y782N | 91 | 95% | 2.66 |
| 437 | Y782P | 97 | 95% | 9.21 |
| 438 | Y782Q | 93 | 95% | 3.26 |
| 439 | Y782R | 94 | 95% | 2.54 |
| 440 | Y782T | 93 | 95% | 4.96 |
| 441 | Y782V | 89 | 91% | 4.54 |
| 442 | Y782W | 91 | 95% | 2.17 |
| 443 | A784W | 86 | 91% | 7.02 |
| 444 | L790P | 85 | 91% | 0.78 |
| 445 | H793D | 93 | 95% | 0.10 |
| 446 | H793E | 90 | 95% | 0.10 |
| 447 | H793F | 87 | 91% | -3.00 |
| 448 | H793G | 87 | 91% | 1.97 |
| 449 | H793K | 91 | 95% | 0.88 |
| 450 | H793L | 89 | 91% | -1.52 |
| 451 | H793M | 87 | 91% | -1.44 |
| 452 | H793P | 95 | 95% | 1.12 |
| 453 | H793R | 87 | 91% | 0.90 |
| 454 | H793T | 89 | 91% | 0.33 |
| 455 | H793W | 92 | 95% | 1.03 |

Table S2

Selected group of *A. thaliana* *ent*-CPS variants indicating threshold values of effect score higher than 85, a probability of at least 91% and ΔΔG_Fold_ lower than -0.50 kcal/mol.

| **Nr** | **Mutation** | **Effect score [-100;100]** | **Probability [%]** | **ΔΔG_FOLD_**  **[kcal/mol]** |
| --- | --- | --- | --- | --- |
| 1 | T114F | 88 | 91% | -0.51 |
| 5 | D336L | 87 | 91% | -0.74 |
| 3 | D377L | 88 | 91% | -1.34 |
| 4 | D377K | 87 | 91% | -0.72 |
| 5 | H391L | 87 | 91% | -0.83 |
| 6 | H391M | 88 | 91% | -0.67 |
| 7 | G422L | 89 | 91% | -2.31 |
| 8 | G422M | 87 | 91% | -2.88 |
| 9 | S597W | 89 | 91% | -1.09 |
| 10 | K778F | 88 | 91% | -1.28 |
| 11 | K778W | 92 | 95% | - 0.90 |
| 12 | H793F | 87 | 91% | -3.00 |
| 13 | H793L | 89 | 91% | -1.52 |
| 14 | H793M | 87 | 91% | -1.44 |

Table S3

Interatomic distances between aa residues of crucial importance for catalytic reaction in six single mutants of *A. thaliana* *ent*-CPS. The mean and standard deviation was applied to calculate the width of confidence intervals. Based on the distance between the data for WT and the mean, the probability (%) that the result is localized beyond the distance k-fold of standard deviation was obtained from the Chebyshev’s inequality.

| **Nr** | **Mutation** | **Distance between atoms [Å]** | | |
| --- | --- | --- | --- | --- |
|  |  | D379:OD2-  GGPS:C15 | D379:OD2-T421:HG1 | D379:OD1- N425:HD2 |
| 1 | WT | 4.02 (k=12, p≤0.006) | 5.53 (k=2, p≤0.25) | 2.12 (k=2, p≤0.25) |
| 2 | T114F | 3.24 | 5.22 | 2.23 |
| 3 | D336L | 3.24 | 5.22 | 2.23 |
| 4 | D377L | 3.32 | 5.23 | 2.26 |
| 5 | G422L | 3.24 | 5.11 | 2.23 |
| 6 | S597W | 3.24 | 5.22 | 2.23 |
| 7 | K778F | 3.24 | 5.22 | 2.23 |
| Mean and standard deviation | | 3.27 ± 0.06 | 5.25 ± 0.13 | 2.21 ± 0.05 |

Table S4

Interatomic distances between aa residues of crucial importance for catalytic reaction in eleven single mutants of *A. thaliana* *ent*-CPS of impaired catalytic properties. The mean and standard deviation was applied to calculate the width of confidence intervals. Based on the distance between the data for WT and the mean, the probability (%) that the result is localized beyond the distance k-fold of standard deviation was obtained from the Chebyshev’s inequality.

| **Nr** | **Mutation** | **Distance between atoms [Å]** | | | |
| --- | --- | --- | --- | --- | --- |
|  |  | D379:OD2-  GGPS:C15 | D379:OD2-T421:HG1 | D379:OD1- N425:HD2 | |
| 1 | WT | 4.02 (k=3, p≤0.11) | 5.53 (k=1, p≤1) | 2.12 (k=1, p≤1) |  |
| 2 | E211A | 3.24 | 5.22 | 2.23 |  |
| 3 | H331A | 3.24 | 5.22 | 2.23 |  |
| 4 | H331R | 3.24 | 5.22 | 2.23 |  |
| 5 | R340A | 3.24 | 5.22 | 2.23 |  |
| 6 | D377A | 3.27 | 6.23 | 6.11 |  |
| 7 | D379A | - | - | - |  |
| 8 | D380A | 3.55 | 5.16 | 2.17 |  |
| 9 | T421A | 3.24 | - | 2.23 |  |
| 10 | T421S | 3.54 | - | 2.21 |  |
| 11 | N425A | 3.54 | 5.08 | - |  |
| 12 | D503A | 3.24 | 5.22 | 2.23 | |
| Mean and standard deviation | | 3.40 ± 0.25 | 5.34 ± 0.35 | 2.60 ± 1.23 | |

Table S5

Interatomic distances between aa residues of crucial importance for catalytic reaction in seven selected double mutants of *A. thaliana* *ent*-CPS of putatively improved catalytic properties. The mean and standard deviation was applied to calculate the width of confidence intervals. Based on the distance between the data for WT and the mean, the probability (%) that the result is localized beyond the distance k-fold of standard deviation was obtained from the Chebyshev’s inequality.

| **Nr** | **Mutation** | **Distance between atoms [Å]** | | | | |
| --- | --- | --- | --- | --- | --- | --- |
|  |  | D379:OD2-  GGPS:C15 | D379:OD2-T421:HG1 | D379:OD1- N425:HD2 | | |
| 1 | WT | 4.02 (k=3, p≤0.11) | 5.53 (k=3, p≤0.11) | 2.12 (k=3, p≤0.11) | |  |
| 2 | T114F/D377L | 3.32 | 5.23 | 2.26 |  |  |
| 3 | T114F/S597W | 3.24 | 5.22 | 2.23 |  |  |
| 4 | D336L/S597W | 3.24 | 5.22 | 2.23 |  |  |
| 5 | D336L/K778F | 3.24 | 5.22 | 2.23 |  |  |
| 6 | D377L/G422L | 3.40 | 5.12 | 2.24 |  |  |
| 7 | D377L/S597W | 3.32 | 5.23 | 2.26 | |  |
| 8 | D377L/K778F | 3.32 | 5.23 | 2.26 | | |
| Mean and standard deviation | | 3.39 ± 0.26 | 5.25 ± 0.12 | 2.23 ± 0.05 | | |
